# Impact of Body Mass Index on Clinical Outcomes of LLETZ

**DOI:** 10.3390/jcm15114307

**Published:** 2026-06-02

**Authors:** Chiara Paternostro, Elmar Joura, Magdalena Postl, Eva Maria Langthaler, Lejla Samson, Sophie Pils

**Affiliations:** 1 Department of Obstetrics and Gynecology, Medical University of Vienna, Spitalgasse 23, 1090 Vienna, Austria; 2 Department of Pathology, Medical University of Vienna, Spitalgasse 23, 1090 Vienna, Austria

**Keywords:** body mass index, obesity, overweight, LLETZ, cervical dysplasia, cervical intraepithelial neoplasia, resection margin, cervical screening, surgical training

## Abstract

**Background/Objectives:** Large loop excision of the transformation zone (LLETZ) is used as a standard treatment for cervical dysplasia. Although obesity is associated with reduced cervical screening participation and may complicate gynecologic procedures, evidence on the impact of body mass index (BMI) on LLETZ outcomes remains limited. This study aimed to evaluate the association between BMI and clinical outcomes after LLETZ, with a particular focus on margin status. **Methods:** This retrospective single-center cohort study included 1397 patients who underwent LLETZ for cervical dysplasia between January 2010 and December 2024 at the Medical University of Vienna. Patients were categorized as normal-weight (BMI < 25 kg/m^2^) or overweight/obese (BMI ≥ 25 kg/m^2^). The primary outcome was margin status. Secondary outcomes included postoperative bleeding, lesion localization, histological findings, HPV status, cone depth, and surgeon status. **Results:** Overall, 949 patients had a BMI < 25 kg/m^2^ and 448 had a BMI ≥ 25 kg/m^2^. Negative margins were achieved in 984 patients (70.4%), with no significant difference between BMI groups. Postoperative bleeding occurred in 53 patients (3.8%) and was significantly less frequent in patients with BMI ≥ 25 kg/m^2^. Each 5-unit increase in BMI was associated with a 38% reduction in the odds of postoperative bleeding. BMI was not significantly associated with HPV status or histological findings. **Conclusions:** BMI was not associated with overall margin status after LLETZ, suggesting that overweight and obesity do not adversely affect short-term surgical outcomes in general. Further prospective studies are needed to evaluate long-term outcomes.

## 1. Introduction

Cervical cancer remains a major global health concern and is the fourth most common cancer as well as the fourth leading cause of cancer-related death among women worldwide, despite the widespread implementation of screening and vaccination programs [1,2,3,4,5,6]. Progression from cervical intraepithelial neoplasia (CIN) to invasive cervical cancer generally occurs over 10 to 15 years [5,7,8,9,10]. Early detection and treatment of high-grade cervical lesions are therefore essential to prevent progression to invasive disease. Large loop excision of the transformation zone (LLETZ) is one of the most commonly used excisional procedures for the management of high-grade lesions and has become standard practice in many gynecologic centers because of its high effectiveness, low morbidity, and favorable balance between diagnostic and therapeutic benefit [11,12,13]. In addition to resection margin status and HPV persistence, several other factors have been identified as independent predictors of residual or recurrent disease after excisional treatment [14]. These include older age, particularly being age 35 years or above, smoking, adenocarcinoma in situ (AIS) histology, a history of prior cervical treatment, and higher-grade lesions at the resection margin, especially when endocervical involvement is present [15,16].

Overweight and obesity is an increasingly prevalent health problem worldwide and is associated with a wide range of perioperative challenges [17,18]. In gynecologic surgery, elevated BMI has been linked to impaired surgical exposure including reduced visualization and a higher risk of selected perioperative complications, particularly thromboembolic events [19,20]. At the same time, findings across different surgical populations have been inconsistent, and some studies have suggested that obesity may not uniformly worsen surgical outcomes. Another important consideration is that obesity may contribute to reduced detection of cervical abnormalities through several mechanisms, including lower participation in cervical screening, higher rates of unsatisfactory smear results, and technical difficulties during examination and sample collection due to impaired cervical visualization [21].

However, the available evidence on the association between overweight/obesity and the treatment of cervical dysplasia remains limited, and data specifically addressing LLETZ are scarce. Against this background, the present retrospective study aimed to assess the impact of BMI on clinical outcomes after LLETZ, with a particular focus on margin status.

## 2. Material and Methods

### 2.1. Patient Population and Study Design

In this retrospective single-center cohort study, 1397 patients who underwent LLETZ for cervical dysplasia during the period of January 2010 to December 2024 at the Department of Obstetrics and Gynecology, Medical University of Vienna were evaluated.

BMI was calculated as weight in kilograms divided by the square of height in meters (kg/m^2^). Patients were classified as normal-weight (BMI < 25 kg/m^2^) or overweight/obese (BMI ≥ 25 kg/m^2^). Obesity was further subdivided into classes I (30–34.9 kg/m^2^), II (35–39.9 kg/m^2^), and III (≥40 kg/m^2^) according to WHO criteria. We used BMI ≥ 25 kg/m^2^ as the primary categorical cut-off because it represents the established threshold for overweight and allowed assessment of elevated BMI more broadly, rather than obesity alone. In the multivariable logistic regression analysis, BMI was included as a continuous variable. Postoperative bleeding was defined as an unscheduled emergency presentation to the outpatient clinic due to postoperative bleeding from the operative site within eight weeks after LLETZ, requiring further treatment such as application of topical hemostatic agents, placement of sutures or electrocoagulation either in an out- or inpatient setting. All bleeding events were clinically verified by a consultant in the outpatient clinic.

The histological results of the specimens were divided into the following groups: negative (i.e., no evidence of dysplasia), low-grade intraepithelial lesions (LSIL), high-grade intraepithelial lesions (HSIL), adenocarcinoma in situ (AIS), squamous cell carcinoma (SCC) and adenocarcinoma (AC). Margin status of the LLETZ specimen was divided into two categories: negative margin or positive margin. Specimens with unclear margin status were categorized as having positive margins. During the study period, all specimens were evaluated and graded by eight pathologists specialized in gynecological pathology. Cone depth was defined as distance between the ectocervical and the endocervical margin and the dimension of the cone was assessed in centimeters by specialized pathologists. As intraoperative measurements of the specimen were not available, potential tissue shrinkage during the pathological processing could not be assessed.

TZ was reported as type 1 (squamocolumnar junction completely visible), type 2 (squamocolumnar junction partially visible), and type 3 (squamocolumnar junction not visible) [22]. Hr-HPV genotypes were diagnosed using cobas^®^ HPV test (Roche Molecular Systems, Inc., Pleasanton, CA, USA) and described individually (HPV 16 and 18) or in a pooled category reported as “other hr-HPV” (HPV 31, 33, 35, 39, 45, 51, 52, 56, 58, 59, 66 and 68). Surgeon status was defined as consultant (board-certified specialist) or resident (physician in training). The study was approved by the Ethics Committee of the Medical University of Vienna (EK number 1194/2025) with a waiver of the requirement for informed consent because of the retrospective design of the study.

### 2.2. Parameters Analyzed

Relevant data were extracted retrospectively from medical records. The aim of this study was to assess the impact of BMI on clinical outcomes following LLETZ. The primary outcome was margin status, and the association with BMI was assessed across subgroups. Secondary outcomes included the incidence of postoperative bleeding, lesion localization (endocervical versus ectocervical), and histological results of LLETZ. Furthermore, the following clinical characteristics were described: smoking status, level of training of the operating surgeon (i.e., resident or consultant), dimension of the cone (depth), TZ type, HPV status and age of the patient at LLETZ. HSIL+ was defined as histological evidence of HSIL, AIS, SCC or AC.

The dichotomized BMI classification was used for the primary analysis to compare patients with a BMI < 25 kg/m^2^ with BMI ≥ 25 kg/m^2^ and to ensure adequate group sizes. In addition, BMI categories according to the World Health Organization classification were used for exploratory descriptive subgroup analyses to assess potential trends across overweight and obesity subclasses. Because of the small number of patients in the higher BMI categories, these subgroup analyses were considered exploratory and no inferential statistical testing was performed for these sub-categories.

### 2.3. Statistical Analysis

Statistical analyses were performed with SPSS v26 (released 2019, IBM Corp., Armonk, NY, USA). Continuous variables were tested for normality using the Shapiro–Wilk test and are presented as median and interquartile range (IQR) due to non-normal distribution. Comparisons between two groups were performed using the Mann–Whitney U test for continuous variables and the chi-square test or Fisher’s exact test for categorical variables, as appropriate. Comparisons across multiple categories were performed using the Kruskal–Wallis test.

Logistic regression analysis was performed to identify independent factors associated with positive margins. Odds ratios (ORs) with 95% confidence intervals (CI) were calculated and for BMI represent the change in odds per one-unit increase. For improved clinical interpretability, ORs were additionally expressed per 5-unit increase in BMI. Subgroup analyses were performed stratified by surgeon status and bleeding event, and an interaction term between BMI and these parameters were included to assess potential effect modification. Complete-case analysis was performed for all multivariable logistic regression models. For each model, only patients with available data for the outcome and all covariates included in that model were analyzed. The number of included and excluded patients is reported accordingly. Furthermore, we assessed missing data descriptively by reporting the number of missing values for each variable (Table 1). A *p*-value < 0.05 was considered statistically significant. Figures were generated using Microsoft Word (version 16.43). An artificial intelligence (AI)-based language model (ChatGPT-5.5, OpenAI) to assist with language editing.

## 3. Results

A total of 1397 patients were included in the analysis, of whom 949 (67.9%) had a BMI < 25 kg/m^2^ and 448 (32.1%) had a BMI ≥ 25 kg/m^2^. Patients with BMI ≥ 25 were significantly older compared to normal-weight patients (median 48 vs. 35 years, *p* < 0.001).

Negative margins were achieved in 984 patients (70.4%), while 413 (29.6%) had positive margins. There was no significant difference in margin status between BMI groups (Table 1, 65.5% vs. 67.0%, *p* = 0.06). Among patients with positive margins, the distribution of ectocervical and endocervical involvement did not differ significantly between BMI groups (Table 1; *p* = 0.27).

Cone depth was comparable between BMI groups with a median depth of 1.3 cm and 1.4 cm, respectively. Postoperative bleeding occurred in 53 patients (3.8%). The incidence of bleeding was significantly lower in patients with BMI ≥ 25 compared to those with BMI < 25 (2.0% vs. 4.6%, *p* = 0.02). Overall, 57.7% of LLETZ procedures were performed by residents, whereas 42.3% were performed by consultants. Smoking prevalence did not differ between BMI groups (44.0% vs. 46.9%, *p* = 0.42). HPV status was available for 1135 patients, and no significant differences were observed between BMI groups for HPV 16 (56.3% vs. 52.7%, *p* = 0.26), HPV 18 (10.3% vs. 10.1%, *p* = 0.26), or non-HPV 16/18 infections (37.4% vs. 39.9%, *p* = 0.43). Histological findings following LLETZ were comparable across groups (*p* = 0.72), with HSIL representing the most frequent diagnosis (74.2%).

When stratified into BMI categories according to the WHO criteria defining overweight as BMI 25–29.9 kg/m^2^, obesity class I (30–34.9 kg/m^2^), class II (35–39.9 kg/m^2^), and class III (≥40 kg/m^2^), clinical characteristics and outcomes were largely comparable across groups (Table 2). Median age and cone depth showed no relevant differences. HPV distribution and smoking prevalence remained consistent across BMI categories without a clear trend. Margin status did not differ meaningfully between BMI groups.

When BMI was analyzed as a continuous variable (Table 3), a higher BMI was significantly associated with a reduced risk of postoperative bleeding. Specifically, each 5-unit increase in BMI was associated with a 38% reduction in the odds of bleeding (OR 0.62, 95% CI 0.43–0.90, *p* = 0.011). In contrast, BMI was not significantly associated with margin status. Although a trend toward lower odds of negative margins was observed with increasing BMI, this did not reach statistical significance (Table 3; OR 0.90, 95% CI 0.80–1.01, *p* = 0.061).

No significant associations were found between BMI and HPV status. This included HPV 16 (OR 0.93, *p* = 0.229), HPV 18 (OR 1.03, *p* = 0.792), and pooled HPV infections (OR 0.99, *p* = 0.903). Age was positively correlated with BMI, indicating that older patients tended to have higher BMI values.

In univariable analysis, BMI was significantly associated with endocervical involvement (OR 1.05 per unit increase, *p* = 0.03). Additionally, a multivariable logistic regression for positive margins overall (Table 4) and positive endocervical margins alone (Table 5) was performed. For these multivariable models, 809 of 1397 patients were included in the complete-case analysis. A total of 588 patients were excluded because of missing values in at least one model variable. The association found in univariate analysis was not maintained after adjustment in multivariable analysis (Table 4 and Table 5) and BMI was not significantly associated with the outcome.

In multivariable logistic regression for positive margins, HSIL+ was independently associated with increased odds of positive resection margins (OR 3.49, 95% CI 1.31–9.29, *p* = 0.01). Greater cone depth was associated with reduced odds of positive margins (OR 0.72, 95% CI 0.52–0.98, *p* = 0.03). No significant associations were observed for BMI, age, smoking, HPV subtype, LLETZ performed by a consultant, or TZ 3.

Increasing age and TZ 3 were independently associated with endocervical involvement in patients with positive margins (Table 5: OR 1.04, 95% CI 1.01–1.06, *p* < 0.01; OR 2.94. 95% CI 1.72–5.03, *p* < 0.01). Furthermore, BMI (OR 1.03, *p* = 0.37) smoking status (OR 1.03, *p* = 0.93) and surgeon status (OR 1.15, *p* = 0.652) showed no significant associations.

In a univariable logistic regression analysis restricted to procedures performed by residents, a higher BMI was associated with increased odds of positive margins (OR 1.04 per BMI unit, 95% CI 1.01–1.07; *p* < 0.01). In an additional multivariable subgroup analysis restricted to procedures performed by residents and adjusted for transformation zone type, higher BMI remained associated with increased odds of positive margins (OR 1.03 per BMI unit, 95% CI 1.00–1.07; *p* = 0.04), but the interaction analysis (BMI and surgeon status; *p* = 0.08) did not reach statistical significance.

## 4. Discussion

In this single-center retrospective study, we evaluated the association between BMI and clinical outcomes in 1397 patients undergoing LLETZ. BMI was not associated with margin status overall or positive endocervical margins. In addition, higher BMI was linked to a significantly reduced risk of postoperative bleeding. However, given the low number of bleeding events, this finding should be interpreted with caution and the association should be regarded as exploratory.

In the evaluated cohort, 448 patients (25.2%) had a BMI ≥ 25 kg/m^2^ and were classified as overweight or obese, of whom 161 (11.5%) met the criteria for obesity (BMI ≥ 30 kg/m^2^). Compared to the global prevalence, where approximately 38% of women are classified as overweight or obese, the proportion observed in our cohort was lower [18,23]. Similarly, the prevalence of obesity alone was lower in our cohort (11.5%) compared to the global estimate of 20.8% among women [24]. This difference may be attributable to the age distribution of the study population, as 83.8% (*n* = 1170) of included women were younger than 50 years. Globally, obesity prevalence among women generally peaks around age 50, although the peak occurs later in some regions, particularly Central/Eastern Europe, Central Asia, and high-income countries [23]. A further explanation for the lower percentage of overweight and obese women in our cohort may be their reduced participation in screening programs. In Austria, 42.4% of women have a BMI of ≥ 25 kg/m^2^, a proportion nearly twice as high as observed in the study cohort. Multiple large studies and meta-analyses show that cervical screening participation decreases as BMI increases [25,26,27]. Compared with women of normal weight, those who are overweight or obese have about 6–21% lower odds of being screened, with the greatest reduction seen in individuals with class III obesity [21,26,28]. Even among screened individuals, women with obesity exhibit higher rates of unsatisfactory cytology and lower detection of precancerous lesions indicating possible underdiagnosis, and highlighting this subgroup as particularly vulnerable [29,30,31]. The relatively low proportion of overweight and obese women in our cohort may reflect differences in screening participation, as suggested by previous studies. However, this interpretation remains speculative, since screening behavior was not directly assessed in the present study. Alternative explanations, including referral bias, center-specific demographic characteristics, differences in the catchment population, and socioeconomic factors, may also have contributed to this finding.

Negative margins were observed at similar rates across BMI groups, with no statistically significant differences. Likewise, the distribution of endocervical and ectocervical involvement did not differ between groups (Table 2). These findings suggest that patients with a BMI ≥ 25 kg/m^2^ did not experience worse short-term surgical outcomes compared to normal-weight individuals. Due to incomplete follow-up data in this study, long-term outcomes, including the prevalence of persistent or recurrent disease after LLETZ in relation to BMI, could not be evaluated. Clarke et al. analyzed 10,614 patients in a large retrospective cohort study, of whom 6.4% developed post-treatment CIN3+. The two-year risk of CIN3+ (including CIN3 and cervical cancer) was highest among patients with obesity (8.7%) compared to those with normal weight (5.6%). A progressive increase in hazard ratios for post-treatment CIN3+ was observed with rising BMI, ranging from 1.19 in overweight women to 1.89 in those with class III obesity (BMI ≥ 40 kg/m^2^). In contrast to our findings, Clarke et al. suggested that higher BMI may be associated with worse long-term outcomes, potentially due to positive margins. Nevertheless, this assumption is not yet supported by clear statistical evidence, and further prospective studies are necessary to clarify potential associations.

Another possible association may be differences in HPV prevalence as well as clearance among women with obesity. Obesity is known to increase susceptibility to certain infections with a 1.7 fold higher risk for severe infections compared to those with normal weight, increasing to approximately 3-fold higher risk of patients with obesity class III [19,32]. Various feasible pathways explaining this mechanism have been described such as chronic low-grade inflammation and immune dysregulation, hyperglycemia supporting pathogen growth or reduced mucus clearance [33]. Liu et al. found that obesity had no significant effect on HPV prevalence, acquisition, or clearance in mid-adult women, although further research on obesity-related immune mechanisms is warranted [34]. Consistent with these findings, Çiçek et al. described that BMI was not independently associated with high-risk HPV infection or related clinical features, suggesting a limited role of obesity on HPV positivity [35]. Likewise, the data demonstrated in this study showed no variation in HPV distribution according to BMI (Table 2).

Regarding postoperative complications, patients with a BMI ≥ 25 kg/m^2^ exhibited a lower incidence of bleeding events compared to those with a BMI < 25 kg/m^2^ (Table 1; 2.0% vs. 4.6%, *p* = 0.02) and each 5-unit increase in BMI was associated with a 38% lower odds of bleeding. The underlying mechanism of this observed association remains uncertain, as no published evidence explains a potential protective effect of higher BMI on postoperative bleeding after LLETZ. Possible explanations, such as differences in surgical technique, electrosurgical settings, tissue characteristics, hemostatic management, and retrospective reporting bias, may also have contributed to this finding; therefore, the observed association should be regarded as hypothesis-generating and requires confirmation in future prospective studies. Sebastian et al. reported a similar trend in minimally invasive bariatric surgery, where patients with a BMI ≤ 40 had a higher risk of bleeding than those with a BMI > 40 after both sleeve gastrectomy (OR 1.22) and gastric bypass (OR 1.16) [36]. A recent study by Barbaresso R. evaluated the impact of BMI on perioperative outcomes in major laparoscopic surgery for benign gynecologic conditions and found that, although higher BMI groups initially showed an increased overall risk of complications compared with non-obese individuals, this difference was no longer significant after adjustment for confounding factors, while postoperative complication rates did not differ between groups [37]. In contrast to these findings, Meyer et al. reported that in women undergoing laparoscopic hysterectomy, higher BMI was associated with more overall and minor complications as well as longer operative time, but overweight and obese patients had a lower risk of major short-term postoperative complications than those with normal BMI [17]. In general, obese patients without metabolic complications may exhibit lower mortality and composite morbidity than normal-weight individuals, a phenomenon often referred to as the “obesity paradox” [38]. However, this apparent protective effect appears not extend generally to patients with a BMI > 40, who show markedly higher complication rates, particularly with respect to pulmonary complications and deep vein thrombosis [20]. Nevertheless, to date, no large studies have specifically investigated the relationship between LLETZ, postoperative bleeding, and obesity, and further research is needed to clarify this association and explore any potential protective effect.

In subgroup analyses, procedures performed by residents were associated with a higher likelihood of positive margins as BMI increased, with the odds rising by 4% for each additional BMI unit (OR 1.04, 95% CI 1.01–1.07; *p* < 0.01). However, as the formal interaction between BMI and surgeon status was not statistically significant, this finding should be interpreted cautiously and considered exploratory only. It may be partly explained by reduced surgical exposure and more challenging operative conditions in patients with higher BMI. Excess adipose tissue may impair visualization of the transformation zone, limit maneuverability, and complicate precise excision, particularly in procedures performed by less experienced surgeons. Consequently, achieving negative margins may be more difficult in this setting. Surgeon training level and experience may influence adverse event rates, although this relationship appears to be complex. Greater attending surgeon experience, reflected by years in practice or higher procedural volume, has been associated with lower rates of intraoperative adverse events and complications, whereas resident involvement has not been shown to increase mortality [39,40].

The major strengths of this study include the large cohort of 1397 patients undergoing LLETZ at a tertiary referral center specialized in gynecologic oncology, the extended study period of 14 years and the consistent histopathological assessment by a small group of specialized gynecologic pathologists. The main limitations of this study are its retrospective design potentially leading to missing data particularly with respect to follow-up and postoperative events including thromboembolic events. In addition, missing data may have introduced selection bias because complete-case analysis included only patients with available data for all variables included in each model. Due to incomplete follow-up data, this study could not assess long-term outcomes. Specifically, persistent or recurrent HPV infection and/or cervical disease after LLETZ could not be evaluated. Therefore, the present results should be interpreted as short-term surgical outcomes and further studies are needed to determine whether BMI has an impact on long-term outcomes. Furthermore, another limitation is that subgroup analyses of patients with severe obesity were limited by the small number of patients in the highest BMI categories. Thus, findings related to BMI subclasses should be interpreted cautiously and considered exploratory. In addition, bleeding events may have been underreported if patients presented to outpatient clinics or hospitals other than our own institution.

## 5. Conclusions

In conclusion, BMI was not associated with margin status in patients undergoing LLETZ. These findings suggest that obesity does not adversely affect overall short-term LLETZ outcomes, but may increase technical difficulty in procedures performed by less experienced surgeons. The comparatively low proportion of women with a BMI ≥ 25 in this study cohort may indicate a potential screening gap within this population, highlighting the need for targeted, national initiatives to ensure inclusive access to cervical screening programs and increase the participation within this subgroup. Further prospective studies are needed to clarify the impact of BMI on long-term outcomes and to better define the role of surgical training in complex patient populations.

## Figures and Tables

**Table 1:** Basic patient characteristics.

Population (*n* = 1397)			BMI < 25 kg/m^2^ (*n* = 949)	BMI ≥ 25 kg/m^2^ (*n* = 448)	*p*-Value
Age, years ^1^		37 (31–45)	*35 (30–44)*	*48 (33–48)*	*<0.01*
Negative margins ^2^		984 (70.4)	684 (72.1)	300 (67.0)	0.06
Positive margins ^2^		413 (29.6)	265 (27.9)	148 (33.0)	
Ectocervical ^2^		310 (75.1)	204 (77.0)	106 (71.6)	0.27
Endocervical ^2^		103 (24.7)	61 (23.0)	42 (28.4)	
Cone depth in cm (median, IQR) ^1^		1.3 (1.0–1.7)	1.3 (1.0–1.7)	1.4 (1.0–1.8)	0.77
Postoperative bleeding ^2^		53 (3.8)	*44 (4.6)*	*9 (2.0)*	*0.02*
Smoking ^2^		449/996 (45.1)	281/638 (44.0)	168/358 (46.9)	0.42
HPV 16 ^2^		627/1135 (55.2)	429/759 (56.3)	198/376 (52.7)	0.26
HPV 18 ^2^		116/1135 (10.2)	78/759(10.3)	38/376 (10.1)	0.26
Non-HPV 16/18 ^2^		434/1135 (38.2)	287/759 (37.4)	150/376 (39.9)	0.43
Transformation zone type ^2^	1	729/1252 (58.2)	505/835 (60.5)	224/417 (53.7)	0.12
	2	285/1252 (22.8)	182/835 (21.8)	103/417 (24.7)	
	3	238/1252 (19.0)	148/835 (17.7)	90/417 (21.6)	
Histological diagnosis LLETZ ^2^	negative	96 (6.9)	65 (6.8)	31 (6.9)	0.72
	LSIL	146 (10.5)	104 (11.0)	42 (9.4)	
	HSIL	1036 (74.2)	706 (74.4)	330 (73.7)	
	AIS	39 (2.8)	25 (2.6)	14 (3.1)	
	SCC	67 (4.8)	40 (4.2)	27 (6.0)	
	AC	13 (0.9)	9 (0.9)	4 (0.9)	

Data are presented as ^1^ median (interquartile range) or ^2^ number (frequencies); significant results are shown in italics. Abbreviations used: *n*, number; BMI, Body Mass Index; HPV, human papillomavirus; LLETZ, large loop excision of the transformation zone; LSIL, low-grade squamous lesion; HSIL, high-grade squamous lesion; AIS, adenocarcinoma in situ; AC, adenocarcinoma.

**Table 2:** Basic patient characteristics in overweight and obese patients according to BMI subgroups.

BMI Subgroups	Overweight (*n* = 287)	Obesity Class I (*n* = 104)	Obesity Class II (*n* = 42)	Obesity Class III (*n* = 15)
Age	39.0 (33.5–47.0)	40.0 (33.8–50.2)	36.0 (32.0–41.8)	40.0 (35.5–48.0)
BMI ^1^	27.1 (25.8–28.3)	32.4 (31.1–33.6)	37.0 (35.9–38.6)	42.3 (41.0–43.9)
Postoperative bleeding ^2^ (*n* = 53)	8 (2.8%)	1 (1.0%)	0 (0.0%)	0 (0.0%)
HPV 16 ^2^ (*n* = 627)	131/244 (53.7%)	43/83 (51.8%)	21/36 (58.3%)	5/13 (38.5%)
HPV 18 ^2^ (*n* = 116)	26/244 (10.7%)	6/83 (7.2%)	4/36 (11.1%)	2/13 (15.4%)
Non-HPV 16/18 ^2^ (*n* = 434)	98/244 (40.2%)	36/83 (43.4%)	11/36 (30.6%)	7/13 (53.8%)
Cone depth in cm (median, IQR) ^1^	1.4 (1.0–1.8)	1.4 (0.9–1.6)	1.4 (1.0–1.8)	1.8 (1.5–2.1)
Smoking ^2^ (*n* = 996)	113 /233(48.5%)	37/80 (46.2%)	12/32 (37.5%)	6/13 (46.2%)
Positive margins ^2^	94 (32.8%)	34 (32.7%)	16 (38.1%)	4 (26.7%)
Negative margins ^2^	193 (67.2%)	70 (67.3%)	26 (61.9%)	11 (73.3%)

Data are presented as ^1^ median (interquartile range) or ^2^ number (frequencies); BMI subgroups are defined as: overweight = BMI 25–29 kg/m^2^; class I = BMI 30–35 kg/m^2^; class II = BMI 35–40 kg/m^2^; class III = BMI > 40 kg/m^2^.

**Table 3:** Association of BMI (per 5-unit increase) with clinical outcomes.

Variable	OR (95% CI)	*p*-Value
Postoperative bleeding	*0.62 (0.43–0.90)*	*0.01*
Negative margins	0.90 (0.80–1.01)	0.06
HPV16	0.93 (0.83–1.05)	0.23
HPV18	1.03 (0.85–1.24)	0.79
Non HPV 16/18	0.99 (0.89–1.10)	0.90
Age	1.20 (1.13–1.28)	<0.01

Data are presented as odds ratios (OR) with 95% confidence intervals (CI). Significant results are shown in italics.

**Table 4:** Multivariable logistic regression for positive margins.

Variable	Adjusted OR (95% CI)	*p*-Value
BMI	1.02 (0.99–1.06)	0.18
Age	1.02 (1.00–1.03)	0.09
Smoking	0.92 (0.67–1.26)	0.60
HPV 16	1.53 (0.99–2.35)	0.06
HPV 18	1.53 (0.99–2.35)	0.32
LLETZ performed by a consultant	0.95 (0.68–1.33)	0.76
TZ 3	1.1 (0.72–1.71)	0.65
Cone depth	*0.72 (0.52–0.98)*	*0.03*
HSIL+	*3.49 (1.31–9.29)*	*0.01*

Data are presented as adjusted odds ratios (OR) with 95% confidence intervals (CI). Significant results are shown in italics.

**Table 5:** Multivariable logistic regression for positive endocervical margins.

Variable	Adjusted OR (95% CI)	*p*-Value
BMI	1.03 (0.97–1.08)	0.37
Age	*1.04 (1.01–1.06)*	*<0.01*
Smoking	1.03 (0.57–1.86)	0.93
HPV 16	1.60 (0.86–3.01)	0.14
HPV 18	1.93 (0.77–4.80)	0.16
LLETZ performed by a consultant	1.15 (0.63–2.11)	0.65
TZ 3	*2.94 (1.72–5.03)*	*<0.01*

Data are presented as adjusted odds ratios (OR) with 95% confidence intervals (CI). Significant results are shown in italics.

## Data Availability

The data presented in this study is available on request from the corresponding author.

## Abbreviations

The following abbreviations are used in this manuscript:

AC	Adenocarcinoma
AI	Artificial intelligence
AIS	Adenocarcinoma in situ
BMI	Body mass index
CI	Confidence interval
CIN	Cervical intraepithelial neoplasia
HPV	Human papillomavirus
HSIL	High-grade squamous intraepithelial lesion
IQR	Interquartile range
LLETZ	Large loop excision of the transformation zone
LSIL	Low-grade squamous intraepithelial lesion
OR	Odds ratio
SCC	Squamous cell carcinoma
SPSS	Statistical Package for the Social Sciences
TZ	Transformation zone
WHO	World Health Organization
